# Minimal dose CT for left ventricular ejection fraction and combination with chest-abdomen-pelvis CT

**DOI:** 10.1016/j.ejro.2024.100583

**Published:** 2024-06-25

**Authors:** Martin Weber Kusk, Søren Hess, Oke Gerke, Lone Deibjerg Kristensen, Christina Stolzenburg Oxlund, Tina Elisabeth Ormstrup, Janus Mølgaard Christiansen, Shane J. Foley

**Affiliations:** a Radiography & Diagnostic Imaging, School of Medicine, University College Dublin, Belfield, Dublin 4, Ireland; b Department of Radiology & Nuclear Medicine, University Hospital of Southern Denmark, Esbjerg 6700, Denmark; c Department of Regional Health Research, Faculty of Health Sciences, University of Southern Denmark, Odense M 5230, Denmark; d Department of Nuclear Medicine, Odense University Hospital, Odense 5000, Denmark; e Department of Clinical Research, University of Southern Denmark, Odense 5000, Denmark; f Department of Cardiology, University Hospital of Southern Denmark, Esbjerg 6700, Denmark

**Keywords:** Four-dimensional computed tomography, Heart failure, Systolic, Radiation exposure, Ejection fraction, Cardiac functional imaging

## Abstract

**Objectives:**

This prospective study tested the diagnostic accuracy, and absolute agreement with MRI of a low-dose CT protocol for left ventricular ejection fraction (LVEF) measurement. Furthermore we assessed its potential for combining it with Chest-Abdomen-Pelvis CT (CAP-CT) for a one-stop examination.

**Materials & methods:**

Eighty-two patients underwent helical low-dose CT. Cardiac magnetic resonance imaging (MRI) was the reference standard. In fifty patients, CAP-CT was performed concurrently, using a modified injection protocol. In these, LVEF was measured with radioisotope cardiography (MUGA). Patients >18 years, without contrast media or MRI contraindications, were included. Bias was measured with Bland-Altman analysis, classification accuracy with Receiver Operating Characteristics, and inter-reader agreement with Intra-Class Correlation Coefficient (ICC). Correlation was examined using Pearson's correlation coefficients. CAP image quality was compared to previous scans with visual grading characteristics.

**Results:**

The mean CT dose-length-product (DLP) was 51.8 mGycm, for an estimated effective dose of 1.4 mSv, compared to 5.7 mSv for MUGA. CT LVEF bias was between 2 % and 10 %, overestimating end-diastolic volume. When corrected for bias, sensitivity and specificity of 100 and 98.5 % for classifying reduced LVEF (50 % MRI value) was achieved. ICC for MUGA was significantly lower than MRI and CT. Distinction of renal medulla and cortex was reduced in the CAP scan, but proportion of diagnostic scans was not significantly different from standard protocol.

**Conclusion:**

When corrected for inter-modality bias, CT classifies patients with reduced LVEF with high accuracy at a quarter of MUGA dose and can be combined with CAP-CT without loss of diagnostic quality.

## Highlights

• CT can measure ejection fraction (LVEF) at a quarter of MUGA dose.
• Absolute LVEF bias to MRI was proven but classification accuracy was excellent.
• LVEF can be measured as part of chest-abdomen-pelvis CT, at preserved image quality.
• The combined protocol could be performed within in a normal CT timeslot.
• A one-stop CT protocol for cancer- and carditoxicity monitoring is feasible.

## 1 Introduction

Left ventricular ejection fraction (LVEF) is still considered an important metric of cardiac function, e.g. in monitoring potential cardiotoxic effects of chemotherapy [1]. The gold standard for LVEF is cardiac magnetic resonance (MRI) due to its high temporal resolution and reproducibility [2]. However, cost, examination time, claustrophobia, obesity or pacemakers may limit availability. Biplane echocardiography is fast and without ionising radiation but is prone to operator variability and less suitable for serial measurements [3]. Radioisotope cardiography with multigated acquisition (MUGA) is considered reproducible [2], [4] and routinely used to monitor cardiotoxicity. However, it subjects patients to 4–7 mSv of radiation dose [5].

CT is accurate for LV volumetric measurements [6], [7] but not recommended as a primary modality due to high radiation burden, as radiation needs to be applied throughout the entire cardiac cycle for correct detection of end-diastolic (ED) and end-systolic (ES) phases. According to ICRP report 120 [8], the dose of such a protocol can be up to four times higher than the routinely used prospectively ECG-gated CT coronary angiography (CCTA) with radiation pulsing. No diagnostic reference levels specifically for functional cardiac CT have been located. Still, recent studies reported dose-length-products (DLP) between approximately 500 and 1200 mGycm [9], [10] for effective doses up to 30 mSv, using the most recent conversion factors [11], [12].

There are, however, potential benefits to CT. Firstly, spatial resolution is superior to MRI. CT temporal resolution has improved, and acquisition is highly automated for consistent image quality, with automatic tube current modulation and voltage selection. Many cancer patients undergo regular contrast-enhanced chest-abdomen-pelvis (CAP) CT scans, which can potentially be combined with a low-dose CT LVEF protocol. Fang et al. demonstrated combined CCTA and portal-venous abdominal CT [13] without image quality evaluation. A similar approach to LVEF could provide a one-stop protocol without additional contrast, provided injection strategies are adapted to weight-based CAP CT protocols.

Effective doses below MUGA, down to approximately two mSv, have been demonstrated in functional cardiac CT [14], [15], [16] but without validation against MRI. A previous study demonstrated that significant simulated dose reduction did not result in significant LVEF bias compared to full-dose images [17].

Thus, the purpose of the study is to clinically test the absolute agreement and classification accuracy of a functional CT protocol for LVEF with a mean effective dose below two mSv, using MRI as the reference. The secondary objectives are to test whether such a protocol can be combined with CAP-CT with preserved image quality. A tertiary objective is the comparison of CT to MUGA for LVEF measurement.

## 2 Materials and methods

### 2.1 Ethical considerations

This prospective study was approved by the Regional Committee on Health Research Ethics of the Region of Southern Denmark (approval numbers S-20210094/S-20220031) and was performed according to the principles laid out in the Helsinki Declaration. Information regarding the risks associated with the excess radiation exposure was communicated to the patients, both in writing and orally, based on an estimated mean dose of 8 mSv (2 mSv from low-dose CT and six mSv from MUGA, if applicable). As well as absolute values, the equivalent multiple of local yearly background radiation, as well as percentage excess cancer risk at 5 % per Sv [18] were explained, along with risks and precautions relating to injection of iodinated contrast media. All participants provided written, informed consent, which they were free to withdraw at any time.

### 2.2 Patient cohort

Between March 2022 and February 2023, we consecutively included participants (≥18 years) from two groups at the University Hospital of Southern Denmark, Esbjerg: Group 1 from regional oncology departments, referred for follow-up CAP CT and/or MUGA. Group 2 from regional cardiology departments referred for CCTA of suspected coronary disease or functional evaluation with cardiac MRI. Exclusion criteria were: Pregnancy, contraindications according to international guidelines [19] or previous adverse reactions to contrast media injection, estimated glomerular filtration rate (E-GFR) below 30 ml/min/1.73 m^2^, physical or cognitive impairment affecting patient cooperation (e.g. inability to hold breath or lift arms above the head), metal implants, electronic devices presenting an MRI safety risk or with potential to create image artefacts, severe claustrophobia, dyspnoea, fever or failure to complete MRI safety screening.

### 2.3 Study flow

All patients were subjected to low-dose CT for LVEF measurement (LVEF-CT), with cardiac MRI as reference test. In group 1, patients also underwent MUGA and CAP-CT immediately following LVEF-CT, as described below. Patients initially referred to CCTA underwent LVEF-CT and MRI as additional scans at least two days after CCTA to avoid the effects of premedication and excessive one-time contrast dosage. All reference and index tests were performed within three hours, except in a few cases with a mandatory delay between injections as per ESUR guidelines following supplementary MRI contrast examination [19].

### 2.4 CT protocol

All CT scans were performed without premedication on a Somatom FORCE Dual Source CT (Siemens Healthineers, Germany). We conducted an extremely low-dose ungated, tin-filtered, high-pitch (TurboFLASH) scan from the carina to 1 cm below cardiac contour as previously described [20]. From this, a retrospectively ECG-gated helical scan, with no radiation pulsing, was planned to cover the LV and aortic valve. Parameters are listed in Table 1.

**Table 1:** Functional cardiac CT acquisition and reconstruction parameters for both ultra-lowdose planning scan, as well as the functional scan for LVEF measurement.

	**Plan scan**	**LVEF scan**
**Collimation [mm]**	2x96x0.6	2x96x0.6
**Helical pitch**	3.2	0.2–0.3^a^
**Rotation time [s]**	0.25	0.25
**Quality Reference mAs**	16	20
**(Reference) kVp**	100Sn^e^	90
**Reference CTDI**_vol_**[mGy]**^b^	0.06	3.03
**CAREkV slider level**^c^	-	9
**Recon kernel/ADMIRE**^d^**level**	Br32f/5	Br32f/5
**Slice thickness/increment [mm]**	3/3	1/0.8
**Matrix size [pixels x pixels**	512×512	256×256
**Cardiac phase interval**	-	5 %

a Pitch adapted automatically to heart rate

b Spectral tin filter

c Volumetric CT dose index for ATCM reference patient of 75 kg

d Denoting the level of expected contrast enhancement

e Advanced Model-Based Iterative Reconstruction

Injection protocols were designed to maintain the same contrast media dose as standard CCTA (standalone LVEF measurement) or CAP oncology CT (with dosage relative to patient size).

For LVEF-CT, 60 ml Ultravist (370 mgI/ml, Bayer Healthcare) was injected in the antecubital vein at 6 ml/s, followed by 40 ml saline. Volume and flow were reduced to 50 ml at 5 ml/s in patients with body weight below 50 kg per local protocol. For combined LVEF/CAP-CT, saline bolus was extended to 60 ml at 5 ml/s to avoid excessive opacification of the right heart chambers. An additional contrast bolus was then injected for a total volume of 1.2 ml/kg up to a maximum of 145 ml. The scan was triggered by bolus tracking at 150 HU in the descending aorta. CAP scans started 45 seconds after the LVEF scan from lung apices to two cm below the lesser trochanter. All scan and reconstruction parameters were the department's standard CAP protocol (Supplementary material 1).

### 2.5 Reference test: MRI

We used a 1.5 T MRI (Magnetom Aera, Siemens Healthineers, Germany) with an 18-element phased array surface coil. ECG-gated 2-D balanced steady-state free precession (bSSFP) 8 mm slices with 2 mm interslice gap were acquired from mitral annulus to cardiac apex. Echo time was 1.07 ms, repetition time 40.8 ms, flip angle 50 degrees. Twenty-five temporal frames were acquired per slice over 8–12 heartbeats. Matrix size was 192×192, and Field-of-View (FOV) 412×412 mm.

### 2.6 MUGA scan

Planar MUGA was acquired on a Discovery 670 (GE Healthcare, USA) or Syngula Scintron (Medical Imaging Electronics, Germany) with low-energy/high-resolution collimators and a 140 keV±10 % energy window. Left anterior oblique projections at 32 and 36 degrees were acquired five minutes after intravenous injection of a target activity of 800 MBq [^99 m^Tc]-labelled human serum albumin (Vasculosis, Curium Pharma, France). ECG was acquired with an R-R interval variability threshold of ±15 % and 24 temporal bins. A 64×64 matrix resulted in a pixel size of 3.3×3.3 mm. Acquisition time was ten minutes per projection, doubling if rejected beats exceeded 20 %.

### 2.7 Effective dose estimation

Effective dose (D_eff_) was estimated from scanner-reported DLP, using the equation D_eff,CT_= DLP [mGycm] x 0.026 mSv/mGycm [11]. Similarly, the ICRP conversion factor was used to estimate MUGA dose from administered ^99 m^Tc activity as D_eff__,__MUGA_=activity [MBq] x 0.007 mSv/MBq [21].

### 2.8 Image analysis

#### 2.8.1 MRI

Two cardiologists with three years of cardiac MRI experience used cvi42 (Circle Cardiovascular Imaging, Canada) for LVEF analysis on short-axis images with simplified endocardial contours and papillary muscles included in the LV cavity. Long-axis images were used for reference. As MRI served as the reference standard, studies in which any of the reading cardiologists found LVEF quantification problematic were excluded from analysis. Reasons included severe artefacts related to respiration or highly irregular heartbeats, insufficient MRI signal strength, insufficient coverage of the left ventricle or other MRI-related artefacts.

#### 2.8.2 CT

Two cardiac CT technologists with 16 and 6 years of experience in cardiac CT analysed LVEF scans in Syngo.Via VB50 (Siemens Healthineers, Germany) using two different measurement methods: Threshold-based "blood-volume" (BV) mode, excluding papillary muscles from the LV cavity, and "standard" (ST) mode based on LV contouring, including papillary muscles. CT studies, as the index test, were only excluded if the relevant anatomy was not covered by the scan range, thus compromising LVEF measurement.

**Fig. 1:**
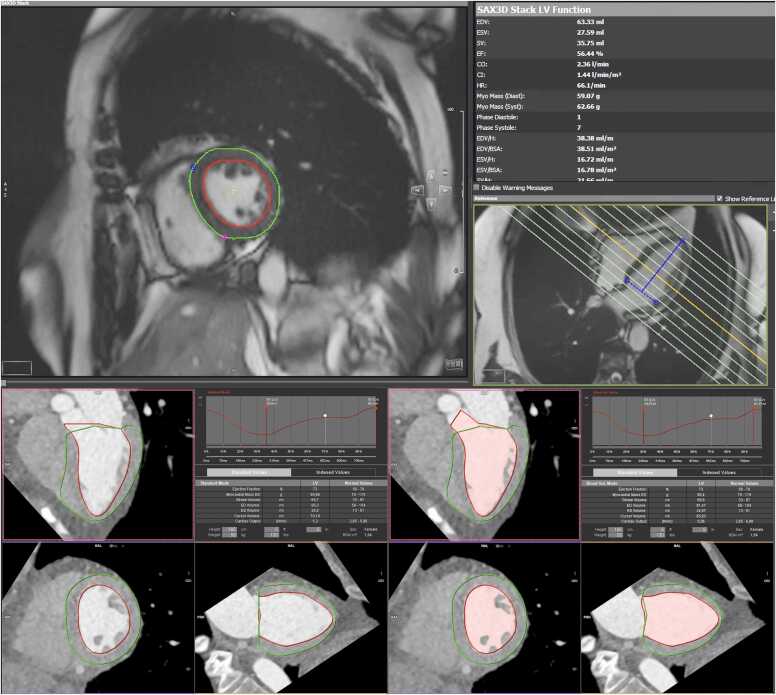
**:**MRI analysis software CVI 42 (top). Bottom left - CT “standard mode” measurement”. Bottom right: CT “Blood Volume” mode, with exclusion of papillary muscles by attenuation thresholding.

The software automatically segmented the LV on MRI and CT (Fig. 1). Readers reviewed and applied corrections as necessary, recording LVEF. End-diastolic, end-systolic and stroke volumes were recorded and indexed to patient body surface area (EDV_i_, ESV_i_ and SV_i,_ respectively) as calculated by the Mosteller equation [22] to account for differences in patient size.

#### 2.8.3 MUGA

Two nuclear medicine technologists with 10 and 18 years of experience analysed MUGA scans using Xeleris 5.0 (GE Healthcare, USA). The software automatically suggested initial ED, ES and background ROIs. These were adjusted as needed, and LVEF was calculated. For MUGA, no exclusion criteria, except equipment malfunction, were applied, this being the current standard of care.

All readers of index- and reference tests were blinded to the results of other readers and modalities. Blinding was assured by creating a completely anonymised dataset copy, unique to each reader and modality, with patient identity replaced by a random three-letter code and all other identifying information removed from the DICOM metadata. Furthermore, readings were undertaken at different time points and/or locations. No clinical information was available to any of the readers.

#### 2.8.4 CAP

**Fig. 2:**
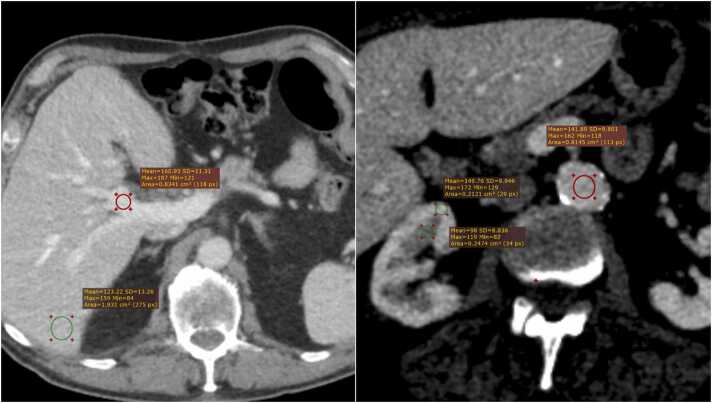
**:**Example of ROI placement for CAP image quality analysis.

CAP scan image quality was compared to the most recent previous scan with standard injection protocol. Attenuation and noise were measured with circular ROIs in the abdominal aorta (level of renal arteries), the main trunk of the intrahepatic right portal vein, liver parenchyma posteriorly in segment 6, renal cortex and medulla of the right kidney (see Fig. 2). Contrast-to-noise ratio (CNR) between liver parenchyma and portal vein and between the renal medulla and cortex was calculated as $\mathrm{CNR}\left(X,Y\right)=2\frac{\mathrm{HU}\left(X\right)-\mathrm{HU}(Y)}{\mathrm{SD}\left(X\right)+\mathrm{SD}(Y)}$ [23], with HU and SD indicating Hounsfield value and pixel standard deviation, respectively, as a measure of visual tissue separation.

Subjective image quality was evaluated by two senior radiologists with 18 and 8 years of oncology CT experience, according to three criteria: Reproduction of hepatic vessels, quality of liver parenchyma enhancement, and the distinction between renal enhancement of cortex and medulla. Images were scored on a five-point scale. The detailed rating scale can be seen in supplementary material 2. These criteria were selected in consensus by the two radiologists as being of prime importance for confident diagnosis.

Readers also provided a binary answer as to the diagnostic acceptability of the scans. 3 mm axial images were reviewed on medical-grade monitors.

The presence of ECG electrodes on the CAP-CT images could identify which studies were performed in conjunction with CT-LVEF. Therefore, images were analysed in two sessions, separated by a 1-week washout period. Each session consisted of a 50/50 per cent mix of experimental/standard protocol studies. A custom-made MATLAB (MathWorks Inc., USA) DICOM-viewer was used, showing the images without any image information. Readers could adjust window/level settings as needed, as well as zooming/panning. The application also allowed automatic recording of image quality ratings. The viewing order was generated randomly for each reader and session.

All study data were recorded in a REDCAP database [24].

### 2.9 Statistical analyses

A minimum sample size of 71 patients was estimated for a power of 80 % and a significance level of 5 % using the procedure described by Lu et al. [25]. The null hypothesis was mean LVEF bias between MRI and CT of 0 % and maximum limits-of-agreement of ±5 % [7].

Continuous variables were presented with means, standard deviations and ranges. For normally distributed variables, two-tailed t-tests or analysis of variance (ANOVA) was used, dependent on the number of factor variables. For ANOVA pairwise post-hoc comparisons, Bonferroni correction was applied. For male/female ratios, the Z-test was used.

All cardiac volumetric measurements performed were aggregated as the mean value obtained by the two readers of each modality.

Absolute agreement between CT and MRI of ESV_i_, EDV_i_ and SV_i,_ as well as LVEF in the entire cohort was assessed with Bland-Altman analysis. Bias and 95 % LoA were calculated, with 95 % confidence intervals (CI) of point estimates. The significance of bias was assessed with a paired t-test. The correlation of CT measurements with MRI was assessed using Pearson's correlation coefficient. For the MUGA subgroup, the same comparisons as above were made for MUGA and CT vs MRI, but only for LVEF, as MUGA does not measure volumes.

We used mixed-effects intraclass correlation coefficients (ICC) to assess LVEF inter-reader agreement for each modality.

Receiver operating characteristics (ROC) were generated using the aggregated LVEF obtained by CT BV, CT ST and MUGA. MRI-derived LVEF was dichotomised according to the 50 % LVEF threshold for the classification of normal/abnormal LVEF to serve as the reference classifier [26]. The area under the ROC curve (AUC) was calculated.

From the reported sensitivities and specificities at each cutpoint, Youden's index was calculated (sensitivity+specificity-1), and the LVEF threshold yielding the maximum value identified for each modality. CT and MUGA LVEF measurements were then dichotomised according to this threshold to produce 2×2 tables, giving sensitivity, specificity and positive/negative predictive values (PPV/NPV). Point estimates were accompanied 95 % CI.

Differences in subjective image quality between CAP scans acquired in combination with CT LVEF scans and previous scans were measured with visual grading characteristics (VGC) using "VGC-Analyzer" [27]. For each image quality criterion, the area under the VGC curve (AUC_VGC_) was calculated, giving a p-value testing the null hypothesis of equivalent quality between protocols. McNemar's test was used to test for significant differences in the number of diagnostic CAP CTs. Reader agreement for image quality scores was measured using Cohen's weighted kappa.

We used STATA 17BE (StataCorp, USA) for all statistical analyses except subjective CAP image analysis. The significance level was 5 %.

## 3 Results

### 3.1 Patient cohort and dose statistics

**Fig. 3:**
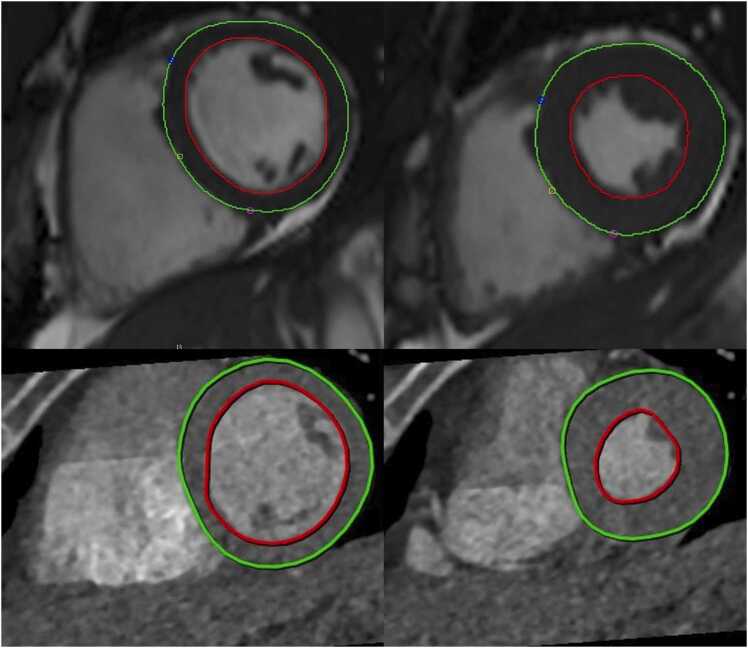
**:**Automated LV segmentations of MRI (top) and CT - standard mode (bottom) in the same patient. ED images are on the left, and ES images are on the right. In this particular case, LVEF was 70 % for MRI and 75 % for CT.

82 patients were scanned: 52 from group 1 and 30 from group 2. No adverse events were recorded from either index or reference tests. In group 1, MUGA was cancelled in three patients (equipment malfunction); thus, only 49 patients had MUGA scans. A representative example from MRI and CT LVEF is shown in Fig. 3.

Four studies were excluded from analysis with compromised MRI image quality (motion), and in one patient, the LV was not completely covered by CT. Demographic and dose details of patients included in the final analysis are shown in Table 2. Mean dose was significantly lower than MUGA, as well as our target value of 2 mSv (p<0.001 in both cases). The plan scan contributed 0.04±0.03 mSv of the CT effective dose at an average scan length of 14.8±2.1 cm, while the LVEF scans covered 10.2±1.5 cm. The difference in examination duration between MRI and MUGA was significant at p=0.012, while CT was significantly shorter than both CT and MUGA (p<0.001), as shown with Bonferroni-corrected ANOVA comparison.

**Table 2:** Characteristics of included patients included in the final analysis. Figures are mean values ± standard deviation, while numbers in parentheses indicate range. CT doses are for LVEF scans and are the combined values of the plan-scan and helical contrast scan. P-values are from unpaired t-test.

	**Combined**	**Group 1**	**Group 2**	**p-value**
**Male/Female ratio**	38/29	22/26	16/13	0.28
**Age [years]**	62.0±11.1 (33−85)	64.8±11.1 (33−85)	59.1±10.7 (35−75)	0.03
**Weight [kg]**	82.2±19.7 (40−146)	81.3±20.8 (40−146)	84.0±16.8 (59−124)	0.54
**Height [m]**	172.9±9.3 (155−200)	173.0±10.2 (155−200)	173.6±7.4 (159−186)	0.76
**BMI [kg/m**^**2**^**]**	27.4±6.1 (16.6–47.3)	27.1±6.6 (16.6–47.3)	27.8±5.0 (20.5–44.5)	0.64
**Heart rate at CT [BPM]**	67.4±13.1(25−119)	69.8±13.2 (45−119)	64.9±13.0 (25−83)	0.09
**Heart rate at MRI [BPM]**	68.0±12.3(41−119)	69.3±14.1 (41−119)	66.7±10.6 (43−89)	0.40
**Heart rate at MUGA [BPM]**	74.6±13.3 (52−103)	74.6±13.3 (52−103)	-	-
**LVEF at MRI [%]**	59.6±10.2 (26−75)	61.3+7.8 (28−75)	56.8±12.9 (26−73)	0.06
**CT duration [min]**	15.70.9±5.6 (7−40)	16.9±6.4 (9−40)	13.8±3.4 (7−23)	0.02
**MRI duration [min]**	28.5±7.4 (17−53)	27.9±6.8 (17−47)	29.6±8.6 (18−53)	0.34
**MUGA duration [min]**	32.4±8.1 (20−57)	32.4±8.1 (20−57)	-	-
**CTDI**_**vol**_**[mGy]**^a^	3.9±2.5 (8–16.6)	4.1±3.01 (0.8–16.6)	3.8±1.6 (1.4–7.1)	0.45
**DLP**^b^**[mGycm]**	51.8±31.9 (10.9–211.8)	55.3±38.1 (10.9–211.8)	49.3±20.3 (17.2–87.6)	0.44
**CT effective dose [mSv]**	1.4±0.8 (0.3–5.5)	1.4±0.9 (0.3–5.5)	1.3±0.52 (0.4–2.3)	0.44
**Tc 99** ^**m**^**activity [mBq]**	811± 20.8 (766−860)	811± 20.8 (766−860)	-	-
**MUGA effective dose [mSv]**	5.7±0.15 (5.3–6.0)	5.7±0.15 (5.3–6.0)	-	-

a Volumetric CT Dose Index

b Dose-Length-Product

### 3.2 Comparison of volumetric measurements

**Fig. 4:**
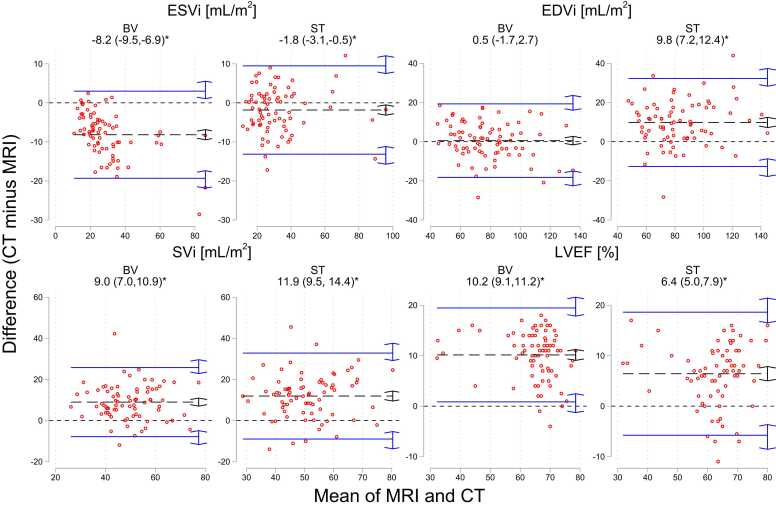
**:**Bland-Altman plots of body-surface-area indexed end-systolic (ESV_i_), end-diastolic (EDV_i_) and stroke volumes (SV_i_), and LVEF between MRI and the two CT measurement modes (BV and ST). Mean bias and upper/lower 95 % limits of agreement (in parentheses) are listed for each comparison and represented by black dashed and blue solid lines respectively. Arrows indicate 95 % confidence intervals of respective point estimates and asterisks denotes bias significantly different from zero, as assessed by the t-test.

**Fig. 5:**
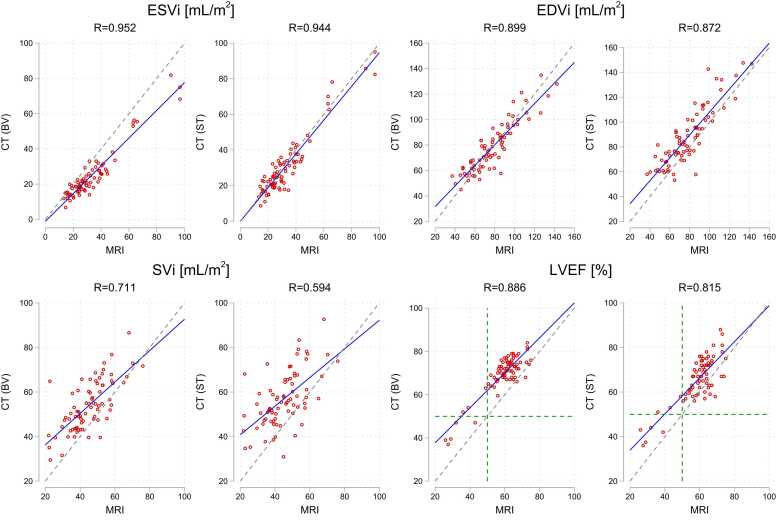
**:**Correlation of body-surface-area indexed end-systolic (ESV_i_), end-diastolic (EDV_i_) and stroke volumes (SV_i_), and LVEF between MRI and the two CT measurement modes (BV and ST). Blue line indicates linear regression fit, compared to the dashed line of perfect concordance. Green dashed line indicate 50 % LVEF. Pearson Correlation Coefficient (R) is reported above each subgraph.

Table 3 lists the absolute indexed volumetric measurements and LVEF for all included patients derived from MRI and the two CT measurement modes. Bland-Altman plots illustrating bias and dispersion of the CT measurements versus the reference of MRI are shown in Fig. 4, showing statistically significant bias between CT and MRI in all except EDV_i_ in the CT BV mode (p=0.630). The corresponding correlations are illustrated in Fig. 5. All pairwise comparisons after ANOVA resulted in p-values <0.001.

**Table 3:** Absolute volumetric measurements by MRI and the two CT measurement modes (“Blood Volume” (BV) and “Standard” (ST)). ESV_i_, EDV_i_ and SV_i_ are end-systolic, end-diastolic and stroke volumes indexed to body surface area, respectively while LVEF is left ventricular ejection fraction. Numbers are means ± standard deviation.

	**ESV**_**i**_**[ml/m**^**2**^**]**	**EDV**_**i**_**[ml/m**^**2**^**]**	**SV**_**i**_**[ml/m**^**2**^**]**	**LVEF [%]**
**MRI**	33.0±17.2	78.1±21.9	44.9±11.3	59.6±10.2
**CT (BV)**	24.8±14.4	78.7±19.7	53.8±11.2	69.7±9.4
**CT (ST)**	31.2±17.3	87.9±23.2	56.8±12.3	66.0±10.2

**Fig. 6:**
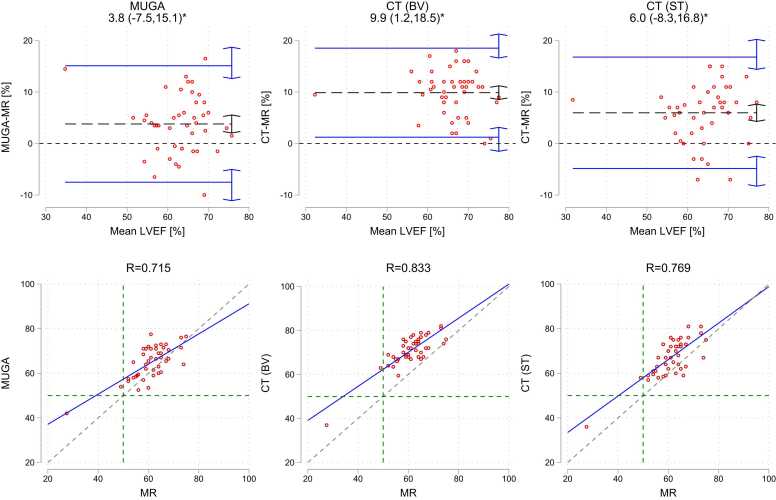
**:**CT and MUGA LVEF Bland-Altman analysis and correlation with MRI, calculated only for the subgroup of patients who underwent MUGA. R= Pearson Correlation Coefficient. Mean difference (bias) and upper/lower 95 % limits of agreement are shown in dotted black and solid blue lines respectively. Arrows indicate 95 % confidence intervals of point estimates. Blue line indicates linear regression fit, compared to the dashed line of perfect concordance. Green dashed line indicate 50 % LVEF.

In Fig. 6, Bland-Altman analysis and LVEF correlations with MRI are illustrated for the subgroup of patients having undergone MUGA. ANOVA revealed that mean LVEF measures were significantly different between all modalities/measurement modes, with pairwise comparison yielding p-values from <0.001–0.002.

MRI-derived LVEF did not differ significantly between groups 1 and 2 as assessed by the t-test (p=0.061).

### 3.3 Reader agreement for LVEF

LVEF inter-reader agreement measured with ICC and 95 %CI were 0.98(0.96–0.98) for CT BV mode and 0.99 (0.99–1.00) for the ST mode when measured in the entire cohort. MRI readers performed similarly with ICC=0.96 (0.99–1.00).

ICC for MUGA was only modest at 0.62 (0.43–0.77), but no significant LVEF bias between readers (p=0.121) as assessed by paired t-test. Calculated for the same subgroup of patients, MR, CT BV and CT ST ICC achieved values of 0.96 (0.93–0.98), 0.99 (0.98–1.00) and 0.99 (0.98–1.00), respectively.

The non-overlap of 95 % CI indicates significant inferiority of MUGA in this regard.

### 3.4 Classification accuracy for reduced LVEF

Of the entire cohort, eight patients had LVEF below 50 %, as classified by MRI. In the subgroup with MUGA, this was only the case for two patients.

Both CT modes had a sensitivity and specificity of 62.5 % and 100 %, respectively (three false negative cases, no false positives) for classifying patients with reduced LVEF (below 50 %) for further heart failure workup. The results of ROC analysis, with recalculated diagnostic measures according to optimal cutpoints, are listed in Table 4. The areas under the ROC curve were not significantly different between BV and ST modes (p=0.323), indicating comparable accuracy.

**Table 4:** Results of ROC analysis of CT (both measurement modes) and MUGA, using mean MRI LVEF of 50 % as reference variable for classification of reduced ejection fraction. Calculations were are for the entire cohort, as well as the subgroup in which MUGA was performed. Diagnostic accuracy measures are computed using LVEF cutoffs at the maximum value of the Youden Index (J_max_). Numbers in parentheses indicate 95 % confidence intervals.

**Index test**	**AUC**^a^	**LVEF@** **J**_**max**_^b^**(%)**	**Sensitivity(%)**	**Specificity(%)**	**PPV**^c^**(%)**	**NPV**^d^**(%)**
**Entire cohort**						
**CT(BV)**^e^	0.99 (0.99–1)	63	100 (63.1–100)	98.5 (92.1–100)	88.9 (51.8–99.7)	100 (94.6–100)
**CT(ST)**^f^	0.99 (0.97–1)	56	87.5 (47.3–99.7)	97.1 (89.8–99.6)	77.8 (40.0–97.2)	98.5 (92.0–100)
**Subgroup of patients with MUGA**						
**CT(BV)**^e^	0.99 (0.96–1)	64	100 (15.8–100)	97.6 (87.4–99.9)	66.7 (9.4–99.2)	100 (91.4–100)
**CT(ST)**^f^	0.98 (0−93−1)	59	100 (15.8–100)	95.2 (83.8–99.4)	50 (6.7–93.2)	100 (91.2–100)
**MUGA**	0.97 (0.91–1)	57	100 (15.8–100)	92.3 (80.5–98.5)	40 (5.3–85–3)	100 (91.0–100)

a Area under the Receiver Operating Characteristic curve

b Maximum value of the Youden Index

c Positive Predictive Value

d Negative Predictive Value

e Blood Volume mode

f Standard mode

### 3.5 CAP image quality

**Fig. 7:**
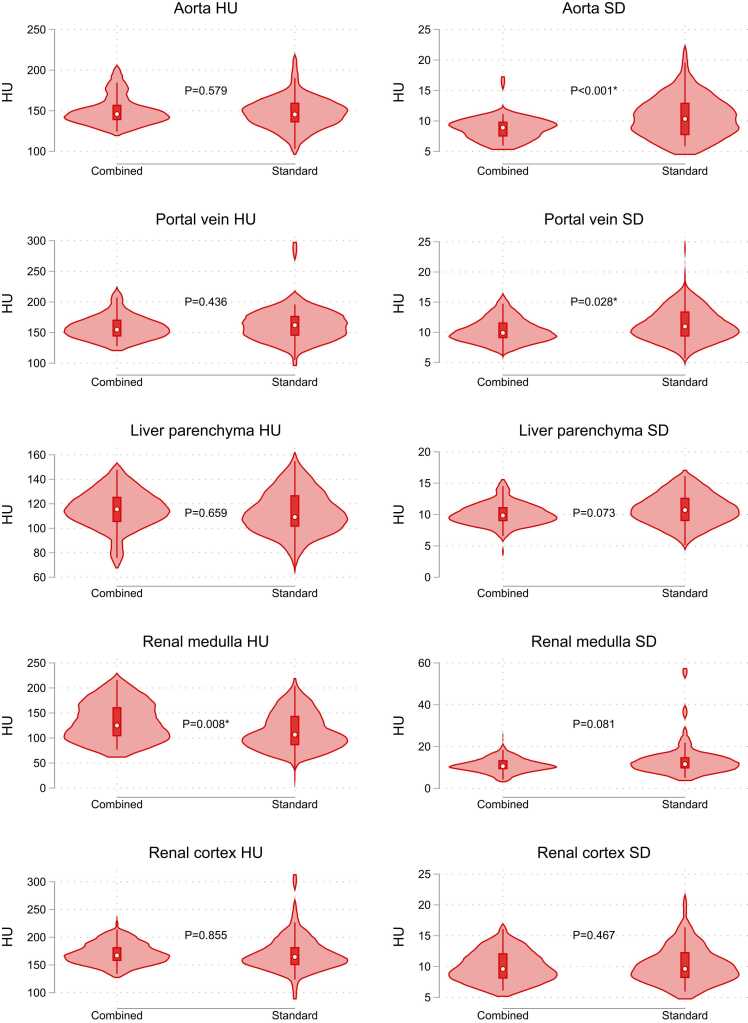
**:** Violin plots of attenuation (HU) and noise (SD) ROI measurements in the combined CT-LVEF/CAP protocol, as well as the previous scans performed with the standard injection protocol. P-values are results of paired t-test for difference in means.

**Fig. 8:**
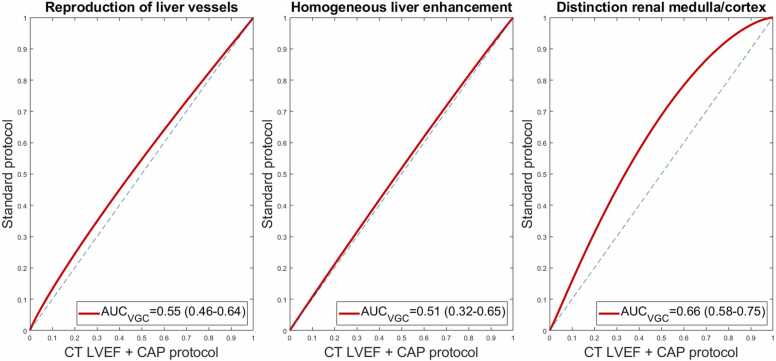
**:** Result of Visual Grading Characteristics analysis from “VGC-Analyzer”. The areas under the VGC-curves (AUC_VGC_) are shown for each of the three subjective image criteria, with 95 % confidence intervals in parentheses. Asterisk denotes AUC significantly different from 0.5 (equivalent image quality assessment).

The results of ROI measurements are visualised in Fig. 7 and tabulated in Table 5. Noise in the aorta and portal vein was significantly lower in the combined protocol by 2.1 and 1.2 HU, respectively (p<0.001 and p=0.031). In the combined protocol, renal medulla mean attenuation was 20.7 HU higher than the standard protocol. As all other measurements were not significantly different, this led to a lower contrast-noise ratio between renal medulla/cortex (3.6±3.1 vs 5.4±3.8, p<0.001). AUC_VGC_ was significantly different only for the criterion related to visual distinction between renal medulla and cortex (p<0.001), but not for the two remaining criteria. VGC-curves, with AUC_VGC,_ are shown in Fig. 8. Cohens' weighted κ was 0.23 for liver vessels, 0.05 for liver enhancement, and 0.20 for distinction of renal medulla and cortex, indicating only slight to fair agreement [28]. One radiologist rated all CAP studies as diagnostic in both experimental and standard CT CAP, while the second found two scans non-diagnostic with the experimental protocol vs. none in the standard protocol, however not statistically significant (McNemar's exact p=0.50). Radiation and contrast media doses were not significantly different between the combined protocol and the previous studies using the standard protocol (p=0.612 and p=0.244).

**Table 5:** Results of ROI measurements of attenuation and image noise, for CT-LVEF/CAP combined scans and previous comparison scans. Contrast-noise-ratio (CNR) is calculated as described in the text. Expressed as means ± standard deviation. Asterisks denote statistically significant differences from paired t-test.

	**CT-LVEF/CAP**	**Standard CT-CAP**	**P-value**
**Aorta HU**^a^	150.9±18.7	146.9±28.2	0.39
**Aorta SD**^b^*	8.8±1.9	10.9±3.5	<0.001
**Portal vein HU**^a^	159.2±20.3	162.9±27.6	0.44
**Portal vein SD**^b^*	10.3±2.1	11.5±3.2	0.03
**Liver HU**^a^	114.5±17.1	112.9±18.8	0.66
**Liver SD**^b^	10.0±2.0	10.8±2.5	0.07
**Liver CNR**^c^	4.55±2.23	4.35±1.96	0.99
**Renal cortex HU**^a^	171.6±21.1	170.6±33.1	0.86
**Renal cortex SD**^b^	12.5±18.7	12.8±17.1	0.95
**Renal medulla HU**^a^*	133.8±38.7	113.1±38.2	<0.001
**Renal medulla SD**^b^	11.4±4.0	13.7±8.2	0.08
**Renal CNR**^c^*	3.6±3.1	5.4±3.8	<0.001

a Hounsfield Units

b Standard Deviation (Image Noise)

c Contrast-to-Noise Ratio

## 4 Discussion

The CT protocol delivered one-quarter the radiation dose of MUGA, which was achieved by a combination of low exposure parameter settings combined with careful scan-range limitation. The protocol by Lee et al. was very similar to ours but applied full-dose in ED or ES phases for coronary diagnosis [14]. Compared to echocardiography, they demonstrated identical bias and LoA but at triple the DLP of ours, using the same scanner. Choi et al. [16] achieved a DLP of 88 mGycm in a comparable population but without a reference standard. It should be noted that these studies calculated effective doses using lower conversion factors, potentially resulting in underestimation [12]. Our study is presumably the first to compare a CT helical protocol without ECG pulsing in ED or ES phases to MRI at this dose level. Although doses could potentially be lowered further by using ECG-pulsing, ectopic beats could result in data loss, as pulsing intervals are determined prospectively from preceding beats, making correct ED/ES phase detection impossible.

Systematic LVEF biases were demonstrated compared to MRI. The LVEF accuracy, as indicated by the width of Bland-Altman LoAs between CT and MRI was comparable to newer studies performed with significantly higher radiation doses [9], [29]. CT and MUGA LVEF LoAs relative to MRI were comparable, indicating similar accuracy.

Mean LVEF bias was 6–10 % dependent on measurement modes compared, but LoA width remained relatively constant. The absolute LVEF differences found between the two CT measurement modes are identical to those demonstrated by Kim et al. [30], using the same software but with images acquired at high (CCTA) dose. Similarly, Schoennagel [31] et al. found a 6 % higher LVEF in MRI when papillary muscles were excluded from the LV volume. Thus, the observed inter-modality bias may be partially explained by different measurement paradigms.

It was noted that the correlation coefficients for LVEF were on the order of those demonstrated when the same MRI studies were measured by different software [32]. Thus, we do not ascribe the differences found in our study to the increased image noise of a low-dose protocol but rather to intrinsic modality or software differences. This is further supported by the high correlation demonstrated between MRI and CT ventricular volumes. Combined with the high classification accuracy, this suggests that CT has the potential to monitor cardiac function, provided inter-modality biases are accounted for. On the other hand, our results highlight the importance of using consistent modalities, software and measurement methods throughout.

EDV_i_ were higher in CT than in MRI. Lower volumes were to be expected when papillary muscles were excluded in the BV mode. Still, these were equivalent to MRI, possibly due to hemodynamic effects of rapid contrast and saline infusion with a transient increase in blood pressure and stroke volume [33], [34]. Comparable studies are often performed in conjunction with CCTA, using beta-blockers and sublingual nitrates, which may offset increased ventricular preload, resulting in lower reported biases. Similar findings to ours were reported in the study by Takx et al. [35] under comparable circumstances. Conversely, the excellent correlation between CT ST measurement and MRI suggests that the lower temporal resolution of CT did not cause ESV_i_ overestimation, which has been cited as a concern [10]. Rather, it can be speculated that the lower spatial (especially longitudinal) resolution of MRI could cause underestimation.

MUGA exhibited lower mean bias, whereas LoA was wider, and inter-reader agreement and MRI correlation were lower than CT, which aligns with the literature [6], [36]. MUGA analyses were assisted by automated LV segmentation, but nevertheless, inter-reader agreement was significantly lower than CT and MRI, which we hypothesise resulted from the markedly lower spatial resolution of MUGA and possibly from the variability of placement of background ROIs.

Image quality was acceptable for combined CAP/LVEF-CT, albeit with a reduced distinction between renal medulla and cortex, likely a consequence of prolonged scan delay due to the adapted injection protocol. This did, however, not affect the overall assessment of image quality. The lower noise shown in the aorta and portal vein ROIs in our experimental protocol can possibly be ascribed to the continued injection during the second pass through the circulatory system, making for more homogeneous attenuation. As the timing of the contrast phases was shifted relative to the standard protocol, it cannot be ruled out that visualisation of, especially, hypervascular lesions could have been compromised. However, as shown previously, in e.g. metastatic colorectal cancer [37], optimal timing of portal-venous enhancement can potentially be predicted by portal vein enhancement of above 120 HU and lower than aortic enhancement, which was achieved as demonstrated in Table 5.

As seen in Table 2, CT can be performed in half the time of both MUGA and MRI, and even when integrated with the CAP protocol, mean examination time was still within a standard CAP timeslot. Thus, in patients referred to both examinations, the total examination time can be reduced to one-third, excluding waiting time. Given availability and sufficient resources, MRI is, of course, preferable, as the patients are not subjected to contrast media administration or ionising radiation. But besides the obvious efficiency gains, implementation of our proposed protocol could potentially simplify coordination of exams, with fewer people and modalities involved, thus reducing the potential for error or variability. Furthermore, replacing MUGA with CT could reduce nuclear medicine staff occupational radiation exposure during preparation, injection and patient handling [38].

### 4.1 Limitations

Our study has obvious limitations. Measurements were only conducted on a single software platform per modality, which may limit generalizability. Different MRI or MUGA acquisition protocols may also influence comparison, which we did not explore. Due to our exclusion criteria, the protocol could not be evaluated in patients who would potentially benefit from it as an alternative to contraindicated MRI, e.g. those with pacemakers. While image noise was high and spatial resolution reduced, compared to CCTA, it cannot be ruled out that large coronary lesions could be visualised from our data. Similarly, the coronary calcium score could potentially be measured using ungated planning scans [39]. However, in the absence of a suitable reference standard, we did not examine these aspects.

Finally, we only examined patients at a single time point. As no independent reference exists, it cannot be determined which modality or measurement methods represent the "true" LVEF. To monitor chemotherapy-related heart failure, the ability to detect clinically significant LVEF changes over time is important. Therefore, our results need to be validated in a longitudinal experiment.

### 4.2 Conclusion

Low-dose functional CT can classify patients with LVEF below 50 %, with excellent diagnostic sensitivity and specificity, albeit with significant absolute bias between 6 % and 10 %, compared to cardiac MRI, dependent on whether papillary muscles were in or excluded in the CT measurement. This can be done at one-fourth of the dose used to measure LVEF with MUGA-scan in half the time and with inter-reader agreement higher than MUGA in the subset of patients undergoing both exams. Adjusting the contrast media injection strategy allowed combined LVEF measurement and CAP CT with preserved image quality at normal weight-based CAP contrast volume. Future studies to test the feasibility of low-dose CT for cardiotoxicity monitoring should include both MRI and CT LVEF measurement over a long follow-up period, with a clinical diagnosis of heart failure as the endpoint.

## Ethics statement

The study was performed in accordance with the principles of the Helsinki Declaration, Danish law and institutional guidelines at the University Hospital of Southern Denmark, Esbjerg. The study was approved by the Scientific Health Research Ethics Committee of the Region of Southern Denmark, approval numbers S-20210094 (group 1, granted March 22–2022), and S-20220031 (group 2, June 7–2022). Patients were provided with written, as well as oral information and were allowed at least 24 hours consideration time, before deciding on participation. Participants provided written informed consent, and were informed that they were free to withdraw at any time, for any reason.

## Funding

The study was funded by grants to the corresponding author (Martin Weber Kusk) by the non-profit research funds listed below:

Esbjerg Fonden (I.C. Møllers Fond)
Radiograf Rådets Forskningsfond
Karola Jørgensens Fond

None of the funders had any influence on the design, conduct, analysis or reporting of the study. None of the authors declare any additional conflicts of interest.

## CRediT authorship contribution statement

**Martin Kusk:** Writing – original draft, Project administration, Funding acquisition, Formal analysis, Conceptualization. **Christina Oxlund:** Writing – review & editing, Formal analysis. **Lone Kristensen:** Writing – review & editing, Formal analysis. **Oke Gerke:** Writing – review & editing, Supervision, Methodology. **Søren Hess:** Writing – review & editing, Supervision, Resources, Conceptualization. **Shane Foley:** Writing – review & editing, Validation, Supervision, Methodology, Conceptualization. **Janus Christiansen:** Writing – review & editing, Methodology. **Tina Ormstrup:** Writing – review & editing, Methodology, Formal analysis.

## Declaration of Competing Interest

The authors declare the following financial interests/personal relationships which may be considered as potential competing interests:Martin Weber Kusk reports financial support was provided by Radiograf Rådets Forskningsfond. Martin Weber Kusk reports financial support was provided by Esbjerg Fonden (I.C. Møllers Fond). Martin Weber Kusk reports financial support was provided by Karola Jørgensens Fond. If there are other authors, they declare that they have no known competing financial interests or personal relationships that could have appeared to influence the work reported in this paper
