# Supplementary material for: Minimal dose CT for left ventricular ejection fraction and combination with chest-abdomen-pelvis CT
Source: Eur J Radiol Open. 2024 Jun 25;13:100583. doi: 10.1016/j.ejro.2024.100583 (PMC11255516; doi:10.1016/j.ejro.2024.100583)
Supplement: Supplementary file 1 — Supplementary material [file mmc1.docx]

Supplementary material 1:CAP acquisition protocol

Standard CT Chest-Abdomen-Pelvis acquisition protocol. Patients are scanned using the Dual-Energy (DE) protocol, images are reconstructed high/low energy mixing ratio of 50% optimized to provide similar attenuation to 120 kVp single energy CT. If the abdominal organs cannot be covered in the 35 cm DE field-of-view, the single-energy protocol is used instead.

|  | **Dual-Energy protocol** | **Single-energy protocol** |
| --- | --- | --- |
| **Tube voltage [kVp]** | 150Sn/80(90) | 120 |
| **Reference time current product [mAs]** | 75/150 | 90 |
| **Helical pitch** | 0.9 | 0.9 |
| **Collimation [mm]** | 2x96x0.6 mm | 2x96x0.6 mm |
| **Rotation time [s]** | 0.5 | 0.5 |
| **Recon kernel** | Br32/Qr32 | Br32 |
