# Supplementary material for: Minimal dose CT for left ventricular ejection fraction and combination with chest-abdomen-pelvis CT
Source: Eur J Radiol Open. 2024 Jun 25;13:100583. doi: 10.1016/j.ejro.2024.100583 (PMC11255516; doi:10.1016/j.ejro.2024.100583)
Supplement: Supplementary file 2 — Supplementary material [file mmc2.docx]

# Supplementary material 2 – Subjective image quality scoring scale

| Criterion | Clear delineation of liver vessels | Liver parenchymal enhancement | Distinction between renal medulla and cortex | Is the scan of diagnostic quality for oncology follow-up? |
| --- | --- | --- | --- | --- |
| Score |  |  |  |  |
| 1 | Not visualized | No visible enhancement | No visible enhancement | Yes |
| 2 | Barely visualized | Insufficient and/or insufficient enhancement | Severely compromised distinction | No |
| 3 | Visualized with some blurring | Suboptimal enhancement and/or homogeneity | Unclear distinction | - |
| 4 | Visualized with slight blurring | Sufficient enhancement and/or homogeneity | Acceptable distinction | - |
| 5 | Sharply visualized | Optimal enhancement and homogeneity | Optimal distinction | - |
