# Supplementary figures and images for: Minimal dose CT for left ventricular ejection fraction and combination with chest-abdomen-pelvis CT
Source: Eur J Radiol Open. 2024 Jun 25;13:100583. doi: 10.1016/j.ejro.2024.100583 (PMC11255516; doi:10.1016/j.ejro.2024.100583)

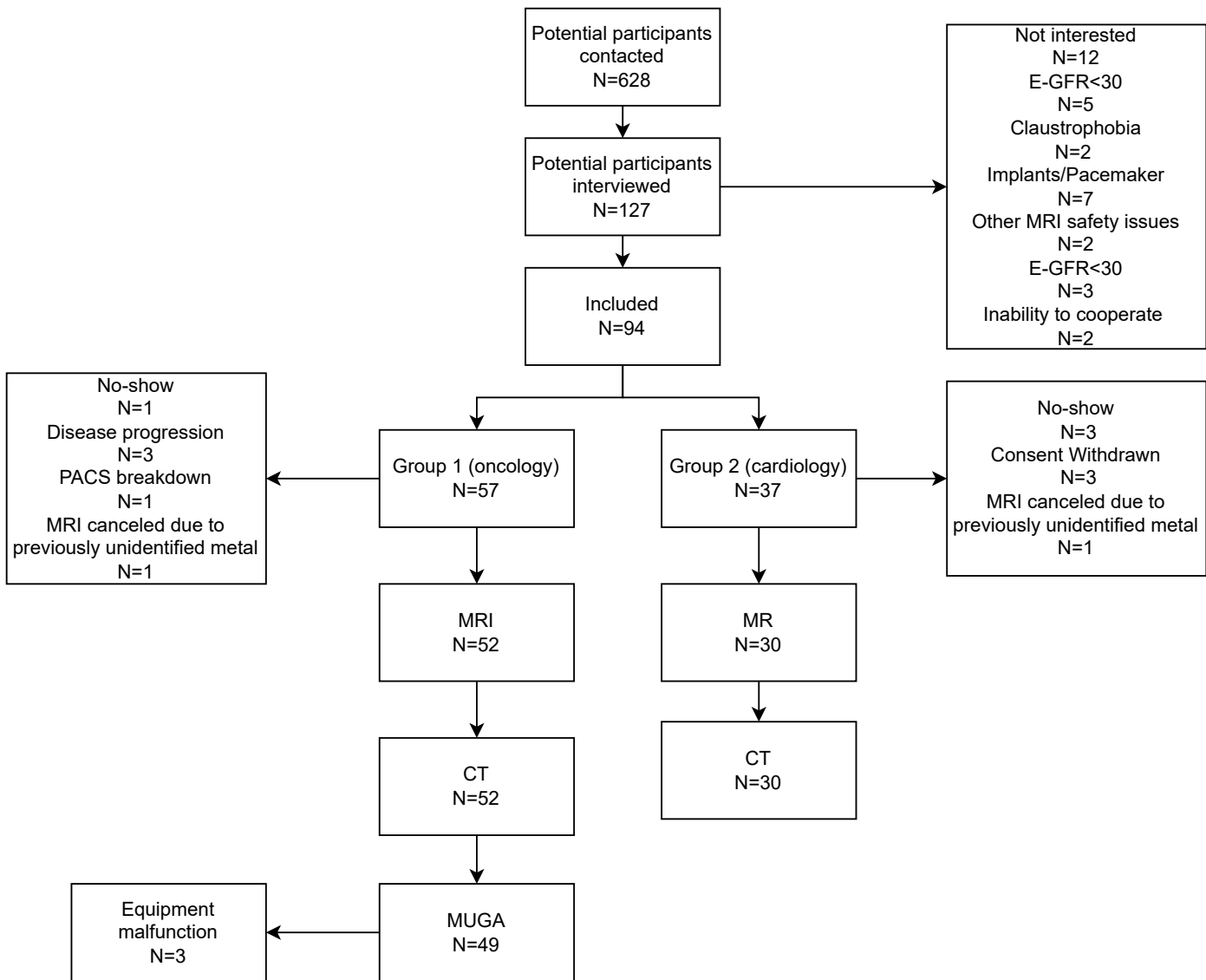

Supplement: Supplementary file 3 — Supplementary material [file mmc3.pdf]
